# Single-Port Robot-Assisted Minimally Invasive Esophagectomy Using the Single-Port Robotic System via the Subcostal Approach: A Single-Center Retrospective Study

**DOI:** 10.3390/cancers17071052

**Published:** 2025-03-21

**Authors:** Jun Hee Lee, Byung Mo Gu, Hyeong Hun Song, You Jin Jang, Hyun Koo Kim

**Affiliations:** 1 Department of Thoracic and Cardiovascular Surgery, Guro Hospital, Korea University College of Medicine, Seoul 08308, Republic of Korea; lee2632@naver.com (J.H.L.); luvotomy7@naver.com (B.M.G.); 2 Department of Medicine, Korea University College of Medicine, Seoul 02841, Republic of Korea; song6540@korea.ac.kr; 3 Division of Upper Gastrointestinal Surgery, Department of Surgery, Guro Hospital, Korea University College of Medicine, Seoul 08308, Republic of Korea; jyjclick@korea.ac.kr

**Keywords:** esophageal neoplasm, esophagectomy, robot-assisted minimally invasive esophagectomy, single port, da Vinci single-port robotic system

## Abstract

Background: Robot-assisted minimally invasive esophagectomy (RAMIE) has gained global popularity. Recent randomized controlled trials have demonstrated that RAMIE results in reduced operative times and a greater number of dissected lymph nodes compared to conventional minimally invasive esophagectomy (MIE). This study provides an initial analysis of single-port (SP) robot-assisted minimally invasive esophagectomy (SRAMIE) using the SP robotic system via the subcostal approach. The primary objective is to examine perioperative outcomes of SRAMIE compared to multi-port RAMIE (MRAMIE) using the Xi robotic system and video-assisted thoracoscopic esophagectomy (VAE). Methods: In this retrospective study, patients who underwent MIE at a single center between February 2017 and December 2024 were analyzed. Patients were divided into SRAMIE (*n* = 17), MRAMIE (*n* = 13), and VAE (*n* = 23) groups. The primary outcome was the incidence of postoperative complications. Secondary outcomes included chest tube duration, length of postoperative hospital stay, postoperative pain levels, and 30-day mortality. Results: The SRAMIE group did not experience conversions to thoracotomy or VAE. Compared with VAE, SRAMIE resulted in significantly shorter chest tube duration (*p* = 0.038), shorter postoperative hospital stays (*p* = 0.036), and lower peak postoperative pain (*p* = 0.003). No significant differences were observed among the groups regarding the total operative time, number of resected lymph nodes, or incidence of postoperative complications. Conclusions: SRAMIE is a feasible approach offering advantages over VAE in recovery and postoperative pain. The comparable perioperative outcomes suggest that SRAMIE may be a viable alternative to conventional MIE, warranting further large-scale studies.

## Simple Summary

The single-port (SP) robotic system, developed for SP surgery, aims to improve patient outcomes by reducing postoperative pain and promoting faster recovery. This system has been approved for thoracic surgery for the first time in the world in South Korea. We report the first case series of SP robot-assisted minimally invasive esophagectomy (SRAMIE) via the subcostal approach. Specifically, we compare short-term perioperative outcomes of SRAMIE with those of multi-port robot-assisted minimally invasive esophagectomy (MRAMIE) and video-assisted thoracoscopic esophagectomy (VAE). The results suggest that SRAMIE is a feasible and equivalent alternative to MRAMIE and VAE, offering reduced postoperative pain and shorter hospital stays compared with VAE. This highlights the potential of SRAMIE in enhancing surgical care for esophageal cancer.

## 1. Introduction

Esophageal cancer is the seventh most commonly diagnosed cancer worldwide and the sixth leading cause of cancer-related mortality [1,2]. Despite advancements in treatment, the five-year survival rate remains below 20% [3]. Current guidelines recommend a multimodal treatment strategy comprising chemotherapy, targeted therapy, immunotherapy, radiation, and surgical intervention to improve survival outcomes. Among these modalities, esophagectomy is considered the most effective treatment for patients with resectable esophageal cancer [4,5].

Esophagectomy has experienced significant advancements over recent decades. Minimally invasive esophagectomy (MIE), introduced in the 1990s [6], has become a standard procedure, offering comparable oncologic outcomes and improved perioperative results than conventional open esophagectomy (OE) [7,8,9,10]. Among MIE techniques, robot-assisted minimally invasive esophagectomy (RAMIE) was introduced in 2003 and has been widely adopted [11]. In 2022, approximately 40% of esophagectomies performed in the United States utilized RAMIE [12]. While the clinical advantages of RAMIE over MIE continue to be investigated, RAMIE provides a superior three-dimensional surgical view and enhanced range of motion via the EndoWrist system, presenting a promising surgical approach [13,14].

The da Vinci single-port (SP) robotic system (Intuitive Surgical Inc., Sunnyvale, CA, USA), designed for SP surgery, features a flexible robotic endoscope and three articulating instrument arms for precise manipulation in confined spaces. SP robotic surgery can offer several benefits, including reduced postoperative pain, improved cosmetic outcomes, and faster recovery [15]. Despite previously reporting the first case of the SP RAMIE (SRAMIE) using the SP robotic system via the subcostal approach [16], its feasibility remains uncertain.

This study compares the perioperative outcomes of SRAMIE using the SP robotic system with multi-port RAMIE (MRAMIE) using the da Vinci Xi robotic system (Intuitive Surgical Inc.), and video-assisted thoracoscopic esophagectomy (VAE). Additionally, a detailed description of the surgical technique is provided.

## 2. Materials and Methods

### 2.1. Study Design and Patient Selection

**Figure 1:**
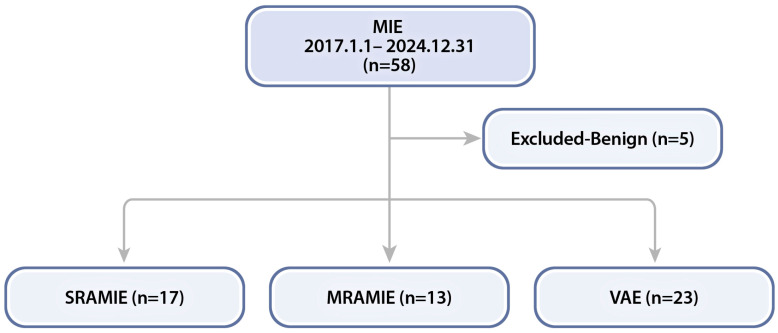
Flowchart of patients who underwent minimally invasive esophagectomy (MIE) at our center. MRAMIE, multi-port robot-assisted minimally invasive esophagectomy; SRAMIE, single-port robot-assisted minimally invasive esophagectomy; VAE, video-assisted thoracoscopic esophagectomy.

This study included 53 patients who underwent MIE for esophageal cancer at Korea University Guro Hospital between February 2017 and December 2024. Patients were categorized into three groups based on the surgical approach: SRAMIE using the da Vinci SP system (*n* = 17), MRAMIE using the da Vinci Xi system (*n* = 13), and VAE (*n* = 23) (Figure 1).

The primary outcome was the incidence of postoperative complications. Secondary outcomes included chest tube duration, length of postoperative hospital stay, postoperative pain, and 30-day mortality. For patients with suspected T3 or T4 or lymph node (LN) involvement, neoadjuvant chemotherapy or chemoradiation therapy was recommended, followed by surgical re-evaluation following induction therapy. Upfront surgery was rarely performed as palliative treatment for dysphagia in patients with cT3N0 disease.

Surgical indications were similar among the three groups and aligned with those of conventional MIE [17,18]. Relative contraindications for SRAMIE included fibrothorax and vascular invasion. In the Republic of Korea, owing to differences in associated patient costs between RAMIE and VAE, the surgical approach (RAMIE vs. VAE) was selected by the patients. MRAMIE was first performed at our institution in April 2017, whereas SRAMIE was introduced in 2022 and has since become the standard approach. All thoracic and cervical procedures were performed by the same thoracic surgeon (H.K.K.), while all abdominal procedures were performed by the same upper gastrointestinal surgeon (Y.J.J.), each serving as the principal surgeon for their respective surgeries. Both surgeons have performed over 1000 cases of minimally invasive surgery.

### 2.2. Perioperative Data

Preoperative assessments included medical history, laboratory tests, esophagography, gastroscopy, endoscopic ultrasonography, chest radiography, computed tomography (CT), positron emission tomography (PET)-CT, and pulmonary function testing. Data on patient and tumor characteristics, as well as intraoperative and postoperative outcomes, were collected. Pathological staging was determined according to the American Joint Committee on Cancer (AJCC) 8th edition TNM staging system [19]. Major postoperative complications were classified based on the Clavien–Dindo classification (≥IIIb) [20], while key postoperative complications were defined according to the Esophagectomy Complications Consensus Group criteria [21]. Postoperative pain was assessed using the visual analog scale at peak intensity and on postoperative days (POD) 0, 1, 2, 3, and 7.

### 2.3. Operative Techniques

Patients in both the SRAMIE and MRAMIE groups underwent hybrid laparoscopic RAMIE, comprising a robotic thoracic phase and laparoscopic abdominal phase, with or without an open cervical phase. General anesthesia with one-lung ventilation was administered, and a nasogastric tube was routinely inserted. The procedural choice was determined by the tumor location and LN involvement. For tumors above the carina or with PET-CT indicating uptake in cervical LNs, the McKeown procedure was preferred. Alternatively, for tumors located below the carina, the Ivor–Lewis procedure was selected. The Ivor–Lewis procedure comprises an abdominal phase followed by a thoracic phase, whereas the McKeown procedure consists of the thoracic, abdominal, and cervical stages. Typically, a two-field lymphadenectomy was performed, reserving three-field lymphadenectomy for cases with suspected cervical LN metastasis or tumors in the upper esophagus [4,22]. The gastric conduit was the preferred organ for esophageal replacement.

#### 2.3.1. Thoracic Phase

Carbon dioxide (CO_2_) gas was maintained at 6–10 mm Hg to achieve a better surgical view. The surgical technique varied depending on the type of surgery performed. SRAMIE was performed via a subcostal approach, whereas MRAMIE and VAE were performed via a transthoracic approach.

**Figure 2:**
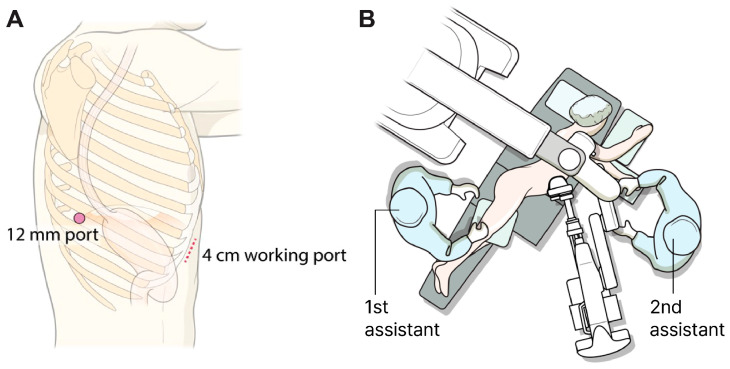
Port mapping and patient position. (**A**) Port mapping for single-port robot-assisted minimally invasive esophagectomy: The 12 mm port served as the assistant port, while the 4 cm working port was used to introduce the robotic system. (**B**) Patient positioning during the thoracic phase of single-port robot-assisted minimally invasive esophagectomy.

In the SRAMIE group, patients underwent single-port plus one port surgery using the SP robotic system while positioned in the lateral decubitus position. A single 4 cm incision was made along the subcostal margin, anterior to the 7th–9th ribs, and a 12 mm assistant port was created at the 8th intercostal space (ICS) along the posterior axillary line (Figure 2A). As previously described, a subcostal tunnel was created via a 4 cm incision [15]. A videoscope was inserted into the thoracic cavity through the 12 mm port with CO_2_ insufflation, facilitating safer and simpler visualization for creating the subcostal tunnel. An SP access port (Intuitive Surgical Inc., Sunnyvale, CA, USA) was installed at the working port and docked to the da Vinci SP patient cart. Subsequently, the first surgical assistant stood on the patient’s right side and inserted an assistant device through the 12 mm assistant port. Meanwhile, a second surgical assistant, positioned on the patient’s left side, inserted another device via the assistant port in the SP access port (Figure 2B).

**Figure 3:**
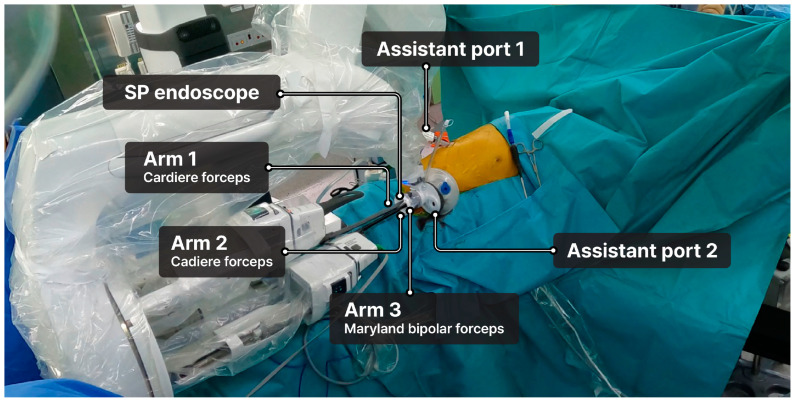
Setup of the da Vinci single-port robotic system.

The SP endoscope was inserted into the central channel, and three robotic arms were installed: arm 1 (Cadiere forceps), arm 2 (Cadiere forceps), and arm 3 (Maryland bipolar forceps or monopolar curved scissors) (Figure 3). The SP system did not include a robotic stapler; therefore, when stapling was required, a surgical assistant inserted the stapler through the assistant port.

**Figure 4:**
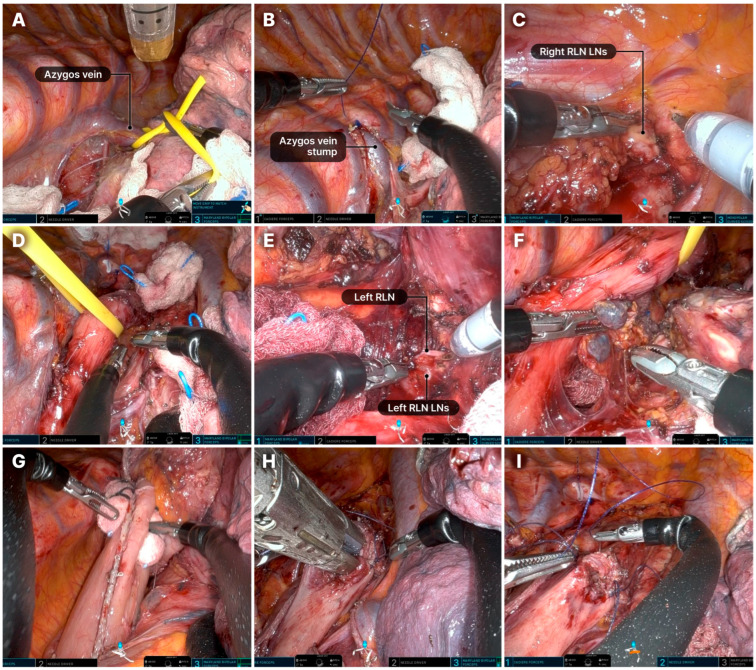
Step-by-step process of the thoracic phase during the Ivor–Lewis procedure. (**A**) Division of the azygos vein with an endostapler. (**B**) The divided azygos vein stump is typically sutured with the chest wall using an absorbable barbed suture (Monofix 4-0). (**C**) Dissection of the upper mediastinum and LNs along the upper thoracic esophagus. The right RLN LNs, as well as LNs from stations 2 and 4, are typically removed. (**D**) Dissection of the upper thoracic esophagus. The thoracic esophagus is encircled with a rubber band and elevated by the assistant. (**E**) Dissection of the left RLN LNs. (**F**) Dissection of the mid and lower thoracic esophagus, with removal of lymph node stations 7–10. (**G**) Gastric conduit is pulled up into the thoracic cavity. (**H**) Posterior wall anastomosis. Partial stapled anastomosis performed in an end-to-side fashion using a linear stapler. (**I**). Anterior wall anastomosis: a two-layer running suture is performed using an absorbable barbed suture (Monofix 4-0). LN, lymph node; RLN, recurrent laryngeal nerve.

The typical sequence of the thoracic phase of the Ivor–Lewis procedure is shown in Figure 4. The surgical sequence did not differ substantially from that of conventional RAMIE [14]. Additional details are provided in the supplementary surgical video (Video S1).

**Figure 5:**
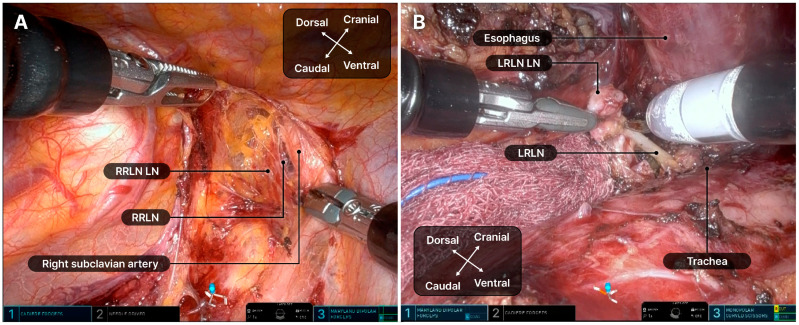
Dissection of the recurrent laryngeal nerve lymph nodes. (**A**) Right recurrent laryngeal nerve lymph nodes. (**B**) Left recurrent laryngeal nerve lymph nodes. LRLN, left recurrent laryngeal nerve; LN, lymph node; RRLN, right recurrent laryngeal nerve.

Dissection of both LNs along the recurrent laryngeal nerve (RLN) is technically demanding and crucial for achieving radical resection (Figure 5). During RLN LN dissection, robotic arm 2 carefully retracted the trachea, while a surgical assistant carefully lifted the esophagus with a rubber band to prevent tearing. A retroesophageal approach was preferred for left RLN LN dissection. If the right bronchial artery significantly obstructed the surgical field, it was dissected using a robotic Hem-o-Lok. Owing to the lack of an energy device, Maryland bipolar forceps were used at a lower power setting to minimize nerve damage, avoiding the main trunk. After surgery, a 20-F chest tube was inserted through the 12-mm port. The subcostal tunnel was closed by suturing the dissected muscle and diaphragm attachment together.

**Figure 6:**
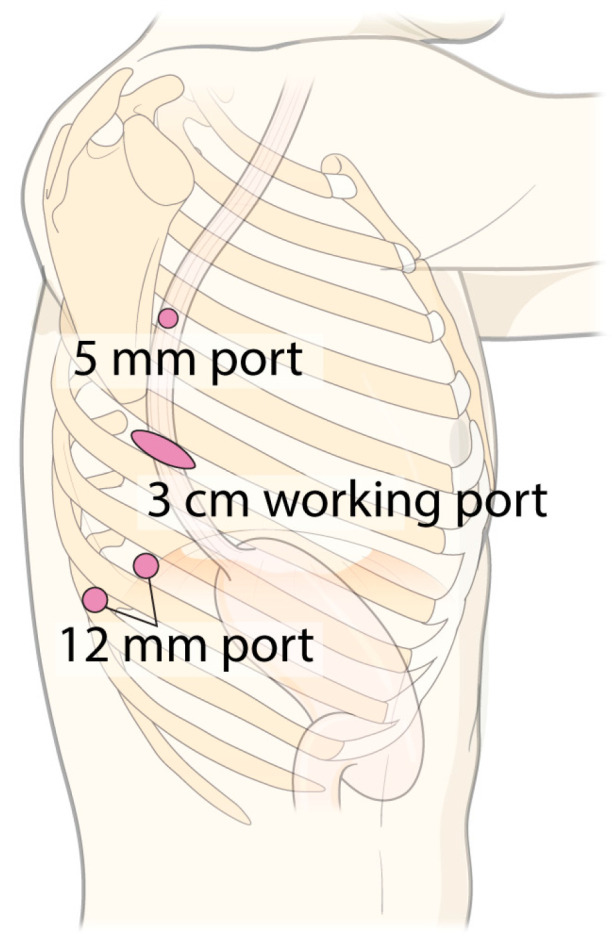
Port mapping for multi-port robot-assisted minimally invasive esophagectomy and video-assisted thoracoscopic esophagectomy.

In the MRAMIE group, patients were positioned semi-prone. Four ports were used: a 3 cm working port with a wound protector (LapSingle Vision; Sejong Medical, Paju, Republic of Korea) at the 6th ICS (arm 3, Xi endoscope), two 12 mm ports at the 8th ICS (arm 1, Maryland bipolar forceps) and 9th ICS (arm 2, Cadiere forceps), and one 5 mm port at the 4th ICS (arm 4, monopolar curved scissors or vessel sealer extension) (Figure 6) [23]. After docking, the surgical assistant stood on the patient’s right side and inserted the device through the assistant port of the multichannel system. The surgical sequence was similar to that used in the SRAMIE group.

In the VAE group, patients were also positioned semi-prone, with four ports positioned similarly to those in the MRAMIE group. The surgeon and scopist stood on the patient’s left and right sides, respectively.

#### 2.3.2. Abdominal Phase

During the abdominal phase, an upper gastrointestinal surgeon performed laparoscopic gastric mobilization and constructed a gastric conduit with LN dissection using a four- or five-port technique. Patients were positioned supine with their legs apart. LNs in the paracardial, left gastric, and celiac regions were dissected. Mobilization of the greater curvature, transhiatal dissection, and conduit creation were performed according to standard surgical protocols [24]. Feeding jejunostomy was performed when a delay in initiating an oral diet was anticipated. Most patients underwent pyloroplasty. A Jackson–Pratt drain was inserted only in cases of severe adhesions or significant bleeding.

#### 2.3.3. Cervical Phase

In the McKeown procedure, a left cervicotomy was performed through a 5 cm incision. After neck dissection and lymphadenectomy, the esophagus was dissected and divided using scissors. The gastric conduit was carefully pulled into the cervical region. After the procedure, a Jackson–Pratt drain was inserted, and the wound was closed.

#### 2.3.4. Anastomotic Technique

Our institution’s esophagogastric anastomotic technique has evolved. Initially, a circular stapler was used for end-to-side anastomosis. This transitioned to a total stapled end-to-side anastomosis with a linear stapler, oversewing the anterior staple line using an absorbable barbed suture, Monofix 4-0 (Hanmi Science Co., Ltd., Seoul, Republic of Korea), in a Lembert fashion. Recently, a partially stapled anastomosis technique was adopted; the posterior wall anastomosis is performed with a linear stapler in an end-to-side fashion, while the anterior wall anastomosis is completed with a two-layer running suture using Monofix 4-0.

#### 2.3.5. Identification of Blood Perfusion Using Indocyanine Green (ICG) Fluorescence Imaging

After gastric tube formation and before anastomosis, blood perfusion in the gastric conduit was assessed via ICG fluorescence imaging using the Rubina^®^ 4K-3D-ICG endoscopic fluorescence imaging system (KARL STORZ SE & Co. KG, Tuttlingen, Germany) or the PINPOINT^®^ endoscopic fluorescence imaging system (Novadaq Technologies Inc., Mississauga, ON, Canada) following an intravenous injection of 12.5 mg of ICG dye (Diagnogreen; Daiichi-Sankyo Co., Ltd., Tokyo, Japan) [25]. When possible, anastomosis was performed in areas with optimal perfusion patterns.

### 2.4. Postoperative Management

Postoperative management was consistent across all groups. Extubation was performed in the operating room after surgery. Patients typically remained in the intensive care unit (ICU) for one day postoperatively, although those with unstable conditions required a longer ICU stay. Intravenous patient-controlled analgesia was routinely used for pain management. Fluoroscopic esophagography with an oral contrast agent was conducted to assess anastomotic leakage introduced on POD 5, after which a clear liquid diet was introduced. The chest tube was removed the day after diet initiation, following confirmation of appropriate drainage color. Patients were discharged once they tolerated oral intake, recovered sufficiently, and expressed readiness for discharge.

### 2.5. Statistical Analyses

All statistical analyses were performed using SPSS software version 27 (IBM Inc., Armonk, NY, USA). Categorical variables were assessed using the chi-square test and presented as numbers (percentages). Continuous variables were evaluated using the Mann–Whitney U-test or Student’s *t*-test and expressed as medians (interquartile ranges). Statistical significance was set at *p* < 0.05.

### 2.6. Ethical Statement

This study was approved by the Institutional Review Board (approval number 2025GR0107; approval date: 13 February 2025). Given its retrospective design and minimal risk to participants, the requirement for informed consent was waived. This study complied with the ethical principles outlined in the Declaration of Helsinki (2013 revision).

## 3. Results

### 3.1. Clinical Characteristics of Patients Who Underwent SRAMIE Using the SP Robotic System

Table 1 summarizes the characteristics of patients who underwent SRAMIE using the SP system. No conversion to VAE or OE was required. With increasing technical experience, console time exhibited a decreasing trend. One patient required reoperation owing to anastomotic leakage following the McKeown procedure. No 30-day mortality was reported.

### 3.2. Comparative Analysis of SRAMIE Using the SP Robotic System, MRAMIE Using the XI Robotic System, and VAE

Baseline patient characteristics were comparable among the three groups, with no statistically significant differences (Table 2). Most patients were diagnosed with squamous cell carcinoma, whereas only one patient in the VAE group was diagnosed with adenocarcinoma. Most tumors were located in the lower esophagus.

Perioperative outcomes are summarized in Table 3. The R0 resection rates in the SRAMIE, MRAMIE, and VAE groups were 94%, 92%, and 91%, respectively. The median total operative time was 465 min [IQR: 430–542 min] in the SRAMIE group, 427 min [IQR: 395–538 min] in the MRAMIE group, and 455 min [IQR: 378–510 min] in the VAE group (*p* > 0.05). There was no statistically significant difference in console time between the SRAMIE (258 min [IQR: 231–279 min]) and MRAMIE (250 min [IQR: 226–346 min]) groups (*p* = 0.613). The esophagogastric anastomotic technique differed significantly among the three groups (*p* < 0.05). Notably, the SRAMIE group had a significantly shorter chest tube duration and postoperative hospital stay than the VAE group (*p* = 0.038 and *p* = 0.036, respectively). Furthermore, peak postoperative pain was lower in the SRAMIE group than in the VAE group (*p* = 0.003). There were no statistically significant differences in postoperative complications among the three groups. Anastomotic leakage occurred in one patient (type III) in the SRAMIE group, one patient (type II) in the MRAMIE group, and four patients in the VAE group, with two classified as type II and two as type III. Moreover, vocal cord palsy occurred in four patients in the SRAMIE group (one with unilateral type I and three with unilateral type II), three patients in the MRAMIE group (all with unilateral type II), and seven patients in the VAE group (three with unilateral type I, three with unilateral type II, and one with bilateral type I).

## 4. Discussion

This report details our first experience with SRAMIE using the da Vinci SP system via a subcostal approach. We also compare the perioperative outcomes between the SRAMIE, MRAMIE, and VAE groups in the largest case study reported to date. The SRAMIE group had a significantly shorter chest tube duration and postoperative length of stay, as well as lower peak postoperative pain than the VAE group.

Esophagogastric anastomosis is a critical step in esophagectomy, with ongoing debate regarding the optimal anastomosis location and technique. Intrathoracic anastomosis is preferred owing to its lower risks of RLN injury and anastomotic leakage [26]. We avoided circularly stapled esophagogastric anastomosis as it is associated with a higher incidence of dysphagia. Postoperative dysphagia affects up to 66% of patients, markedly impacting their quality of life following esophagectomy [27,28,29]. Aiolfi et al. reported that linear stapled anastomosis is associated with a low incidence of anastomotic leakage and stricture, suggesting its potential advantage in optimizing surgical outcomes [30]. Hence, when conduit length permits, we prefer linear stapled anastomosis—totally or partially stapled techniques. Meanwhile, few studies compare these methods directly. Partially stapled anastomosis is technically challenging owing to the requirement for hand-sewn closure on the anterior side but offers improved lumen preservation, reduced tension from imprecise stapling, and prevention of stapler line intersections [31]. Advances in robotic systems that enable precise, efficient, and reproducible suturing have made this approach the preferred method at our center.

Several studies have shown that MIE is superior to OE for short- and long-term oncological outcomes [7,8,9,10]. Recently, the RAMIE and REVATE trials reported that RAMIE results in shorter operative times and more dissected LNs than conventional MIE [32,33]. In the current study, we found SRAMIE comparable to MRAMIE and VAE regarding operative time and the number of dissected LNs. However, the SRAMIE group had a shorter chest tube duration, reduced postoperative stay, and lower peak postoperative pain than the VAE group, with no significant differences observed between SRAMIE and MRAMIE.

The SRAMIE group underwent a subcostal approach during the thoracic phase. Postoperative pain after esophagectomy is largely attributable to intercostal nerve damage caused by the intrathoracic approach. In contrast, extrathoracic approaches, including subcostal and subxiphoid approaches, minimize intercostal nerve damage, reducing postoperative pain [34,35,36]. Our SRAMIE technique utilizes a subcostal approach to minimize intercostal nerve damage, thus reducing pain and promoting faster recovery. Indeed, the SRAMIE group exhibited significantly lower peak postoperative pain than the VAE group. However, only peak VAS differed significantly, suggesting a disparity in extreme pain severity at a particular moment. Hence, while an initial difference was observed in peak pain intensity, the pain levels between the two groups likely converged over time. No significant pain difference was detected on POD 0, likely due to the small patient sample size. A larger sample is expected to show differences on POD 0.

The observed shorter chest tube duration and postoperative hospital stay in the SRAMIE group may be attributed to the minimally invasive nature of the subcostal approach and the potentially reduced intercostal nerve injury associated with the SP robotic system. The absence of significant differences in postoperative complications among the groups may be due to the relatively small sample size, potentially limiting the statistical power to detect minor differences in postoperative outcomes. Furthermore, the single-center setting and retrospective design of this study may have introduced selection bias regarding patient assignment to different surgical approaches. Future research involving larger cohorts and propensity score matching is recommended to further elucidate these findings.

The number of resected LNs in the SRAMIE group was comparable to those reported in previous studies [32,33,37]. A key advantage of SRAMIE is the precise dissection of LNs along both RLNs compared to VAE and OE. Multi-joint instruments, combined with a fully articulating SP endoscope, facilitate precise and effective RLN LN dissection while significantly reducing the risk of nerve injury. Specifically, 3DHD imaging enables the accurate identification of the left RLNs and their surrounding structures. Meanwhile, EndoWrist instruments allow for dissection parallel to the nerve fibers, ensuring meticulous handling and the removal of LNs in complex, confined spaces. Furthermore, the system compensates for tremors, providing stable and controlled movements that enhance the safety and precision of LNs dissection. Although others have reported a lower incidence of vocal cord palsy in the RAMIE group [38,39,40], a similar incidence was observed among the three groups in this study due to aggressive LN dissection.

The SP robotic system requires several improvements for more optimal utilization in esophageal surgery. First, a cannula diameter of 2.8 cm poses challenges in the transthoracic approach, prompting us to develop a subcostal approach. Although the semi-prone position offers several advantages during the thoracic phase of esophagectomy, the SP robotic system requires a lateral decubitus position during the subcostal approach to prevent instrument collision. To facilitate lung and tissue traction in this position, we created an additional assistant port, enabling the insertion of instruments to effectively retract the lungs and surrounding tissues. Second, the SP system currently lacks a robotic stapler. Thus, an endoscopic stapler was inserted through the assistant port to facilitate stapling as needed. Finally, the maximum reach of the robotic instruments from the single-port outlet is approximately 27 cm, which may pose challenges for accessing the upper mediastinum in taller patients. However, our tallest patient was 178 cm, and no significant difficulties were encountered.

Our SRAMIE approach used a subcostal approach, which is uncommonly utilized by thoracic surgeons. Compared to the conventional transthoracic approach, the subcostal method can reduce postoperative pain and promote recovery by preserving intercostal nerves. However, owing to its unfamiliarity, creating the subcostal tunnel can be challenging. Performing blunt dissection under videoscopic guidance within the thoracic cavity allows the surgeon to palpate the trajectory of the subcostal tunnel, making the procedure safer and simpler. Although obesity is not a contraindication for this approach, the initial creation of the subcostal tunnel may be particularly challenging in patients with obesity.

Although SRAMIE is an innovative procedure, other robotic approaches are emerging. The main limitation of the SRAMIE technique at our center is the laparoscopic abdominal phase. Meanwhile, in high-volume centers, total robotic esophagectomy is being implemented, offering potentially superior perioperative outcomes despite a steep learning curve [14,41]. Moreover, transcervical robotic esophagectomy may reduce pulmonary complications but fails to facilitate mediastinal lymph node dissection, a critical aspect of oncologic esophagectomy [42]. Hadzijusufovic et al. reported a new transcervical robotic method using the SP robotic system, warranting further analyses to assess its clinical utility and reproducibility [43].

This study has certain limitations. First, all thoracic and cervical procedures were performed by one thoracic surgeon (H.K.K.), while all abdominal procedures were carried out by one upper gastrointestinal surgeon (Y.J.J.) at a single center. Second, this study was a retrospective analysis with a limited number of patients in each group, introducing selection bias. The choice of surgical approach was influenced by clinical and institutional factors. Additionally, confounding factors, such as differences in patient comorbidities, tumor characteristics, and surgeon experience, may have impacted the results. Third, as the standard approach evolved from VATS to MRAMIE and then to SRAMIE, a time-trend bias was present, potentially influencing outcomes. Finally, this study primarily focused on short-term perioperative outcomes, with no data on long-term oncologic outcomes, such as overall and disease-free survival, owing to the recent adoption of SRAMIE at our institution. Further prospective studies with long-term follow-up are required.

## 5. Conclusions

This study demonstrates that SRAMIE using the SP robotic system is a feasible surgical approach, with perioperative outcomes comparable to MRAMIE and VAE. Notably, SRAMIE presents several advantages over VAE, such as shorter chest tube duration, reduced postoperative hospital stay, and lower peak postoperative pain. These findings suggest that SRAMIE may be a suitable alternative to conventional minimally invasive esophagectomy. Further large-scale randomized controlled trials are required to validate its efficacy and long-term oncological outcomes.

## Figures and Tables

**Table 1:** Summary of patients who underwent single-port robot-assisted minimally invasive esophagectomy using the single-port robotic system.

Sex/Age (Years)	Tumor Location	Diagnosis	Procedure	Console Time (min)	Chest Tube Duration/Postoperative LOS (Days)	Intraoperative/Postoperative Complications
M/55	Middle	SqCC	McKeown	400	6/12	
M/57	Upper	SqCC	McKeown	239	19/43	Re-operation owing to anastomotic leakage, vocal cord palsy, pneumonia
M/68	Middle	SqCC	McKeown	237	11/14	Vocal cord palsy
M/52	Lower	SqCC	Ivor–Lewis	253	9/14	
M/75	Lower	SqCC	Ivor–Lewis	272	6/10	
M/63	Lower	SqCC	Ivor–Lewis	225	7/9	
M/54	Lower	SqCC	Ivor–Lewis	188	7/10	Vocal cord palsy
M/56	Middle	SqCC	Ivor–Lewis	211	7/10	
M/72	Middle	SqCC	McKeown	270	8/16	
M/74	Lower	SqCC	Ivor–Lewis	283	12/18	Pneumonia
M/77	Middle	SqCC	Ivor–Lewis	307	10/13	Pleural effusion
M/67	Upper	SqCC	McKeown	275	19/34	Vocal cord palsy, pneumonia
M/73	Middle	SqCC	McKeown	325	8/14	
F/69	Lower	SqCC	Ivor–Lewis	198	6/8	
M/49	Lower	SqCC	Ivor–Lewis	272	11/53	Seizure
M/62	Lower	SqCC	Ivor–Lewis	240	6/11	Pleural effusion
M/67	Upper	SqCC	McKeown	258	5/11	

MRAMIE, multi-port robot-assisted minimally invasive esophagectomy; SRAMIE, single-port robot-assisted minimally invasive esophagectomy; SqCC, squamous cell carcinoma; VAE, video-assisted thoracoscopic esophagectomy.

**Table 2:** Baseline patient characteristics.

Variable	SRAMIE	MRAMIE	VAE	*p*-Value		
	(*n* = 17)	(*n* = 13)	(*n* = 23)	SRAMIE vs. MRAMIE	SRAMIE vs. VAE	MRAMIE vs. VATS
Age, years	67 [55–72]	64 [61–65]	65 [61–70]	0.713	0.829	0.415
Sex, male	16 (94%)	11 (85%)	21 (91%)	0.565	1.000	0.609
BMI (kg/m^2^)	22.8 [19.7–24.1]	22.4 [19.9–23.8]	21.4 [19.9–24.7]	0.663	0.810	0.827
Comorbidities						
HTN	10 (59%)	8 (61%)	10 (43%)	1.000	0.337	0.298
DM	1 (6%)	4 (31%)	5 (22%)	0.138	0.216	0.693
COPD	1 (6%)	3 (23%)	2 (9%)	0.290	1.000	0.328
ASA	2 [2–3]	3 [2–3]	3 [2–3]	0.462	0.538	0.731
Pathology				1.000	1.000	1.000
SqCC	17 (100%)	13 (100%)	22 (96%)			
AC	0	0	1 (4%)			
Neoadjuvant therapy				0.698	0.233	0.336
CRT	4 (24%)	4 (31%)	4 (17%)			
CT	0	0	4 (17%)			
None	13 (76%)	9 (69%)	15 (65%)			
Tumor location				0.340	1.000	0.204
Upper	3 (17%)	5 (38%)	5 (22%)			
Middle	6 (35%)	5 (38%)	7 (30%)			
Lower	8 (47%)	3 (23%)	11 (48%)			
Operative types				0.713	0.680	0.265
Ivor–Lewis	10 (59%)	6 (46%)	15 (65%)			
McKeown	7 (41%)	7 (54%)	8 (35%)			
Clinical TNM stage				1.000	0.143	0.391
I	10 (59%)	7 (54%)	7 (30%)			
II	3 (18%)	3 (23%)	10 (43%)			
III	4 (23%)	3 (23%)	6 (26%)			
Clinical status				0.103	0.909	0.607
cT1a N0/N1	2 (12%)/1 (6%)	4 (31%)/0	3 (13%)/0			
cT1b N0/N1	8 (47%)/0	3 (23%)/2 (15%)	7 (30%)/2 (9%)			
cT2 N0	4 (24%)	2 (15%)	5 (22%)			
cT3 N0/N1/N2	2 (12%)/0/0	0/2 (15%)/0	3 (13%)/1 (4%)/1 (4%)			
cT4 N0	0	0	1 (4%)			

AC, adenocarcinoma; CRT, chemoradiotherapy; CT, chemotherapy; MRAMIE, multi-port robot-assisted minimally invasive esophagectomy; SRAMIE, single-port robot-assisted minimally invasive esophagectomy; SqCC, squamous cell carcinoma; VAE, video-assisted thoracoscopic esophagectomy. Data are expressed as numbers (%) or medians [interquartile range].

**Table 3:** Perioperative outcomes.

Variables	SRAMIE	MRAMIE	VAE	*p*-Value		
	(*n* = 17)	(*n* = 13)	(*n* = 23)	SRAMIE vs. MRAMIE	SRAMIE vs. VAE	MRAMIE vs. VAE
Thoracotomy conversion	0 (0%)	1 (8%)	1 (4%)	0.433	1.000	1.000
R0 resection	16 (94%)	12 (92%)	21 (91%)	1.000	1.000	1.000
Total operative time (min)	465 [430–542]	427 [395–538]	455 [378–510]	0.187	0.389	0.877
Console time (min)	258 [231–279]	250 [226–346]		0.613		
Anastomosis technique				0.002	0.003	0.020
Linear stapling						
Totally stapled	3 (18%)	8 (62%)	3 (13%)			
Partially stapled	13 (76%)	2 (15%)	7 (30%)			
Circular stapling	1 (6%)	3 (23%)	12 (52%)			
Handsewn	0	0	1 (4%)			
Number of LNs resected						
Total LNs	32 [26–42]	30 [20–35]	27 [20–37]	0.240	0.404	0.714
Left RLN LNs	2 [2–4]	2 [1–3]	1 [1–3]	0.265	0.072	0.668
Right RLN LNs	3 [2–4]	2 [1–3]	2 [1–4]	0.207	0.655	0.512
Chest tube duration (days)	7 [6–11]	8 [7–11]	11 [8–16]	0.406	0.019	0.082
Postoperative LOS (days)	13 [10–17]	15 [13–18]	18 [14–40]	0.214	0.030	0.228
Postoperative pain (VAS)						
Peak	4 [3–5]	6 [3–7]	6 [5–7]	0.459	0.003	0.255
POD 0	3 [3–5]	3 [3–7]	5 [3–7]	0.349	0.108	0.928
POD 1	3 [3–4]	3 [3–5]	3 [3–5]	0.703	0.286	0.622
POD 2	3 [2–3]	3 [2–6]	3 [3–5]	0.436	0.557	0.798
POD 3	3 [2–3]	3 [2–3]	3 [2–3]	0.910	0.919	1.000
POD 7	2 [1–3]	3 [1–3]	2 [1–4]	0.420	0.332	0.992
Postoperative complications						
Major complications	2 (12%)	1 (8%)	5 (22%)	1.000	0.677	0.385
Anastomotic leakage	1 (6%)	1 (8%)	4 (17%)	1.000	0.373	0.634
Vocal cord palsy	4 (24%)	3 (23%)	7 (30%)	1.000	0.725	0.709
Pneumonia	3 (18%)	4 (31%)	7 (30%)	0.666	0.471	1.000
Reoperation	1 (6%)	1 (8%)	2 (9%)	1.000	1.000	1.000
Pathological status				0.904	0.543	0.916
pT0 N0/N1	3/0	2/1	2/1			
pT1a N0/N1	3/0	3/1	2/0			
pT1b N0/N1/N3	6/1/1	3/1/0	4/1/0			
pT2 N0/N1	0/0	0/0	2/1			
pT3 N0/N1/N2/N3	1/2/0/0	0/1/1/0	1/2/3/1			
pT4a N3	0	0	1			

LOS, length of stay; LNs, lymph node; POD, postoperative day; RLN, recurrent laryngeal nerve; SRAMIE, single-port robot-assisted minimally invasive esophagectomy; VAE, video-assisted thoracoscopic esophagectomy. Data were expressed as numbers (%) or medians [interquartile ranges].

## Data Availability

The data underlying this article will be shared upon reasonable request from the corresponding author.

## Supplementary Materials

The following supporting information can be downloaded at https://zenodo.org/records/14876942 (accessed on 16 Febrary 2025): Video S1: Surgical video of single-port robotic-assisted minimally invasive esophagectomy using the da Vinci single-port robotic system via the subcostal approach.

## Abbreviations

The following abbreviations are used in this manuscript:

ICG	Indocyanine green
ICS	Intercostal space
LN	Lymph node
MIE	Minimally invasive esophagectomy
MRAMIE	Multiport robot-assisted minimally invasive esophagectomy
OE	Open esophagectomy
PET	Positron emission tomography
RAMIE	Robot-assisted minimally invasive esophagectomy
RLN	Recurrent laryngeal nerve
SP	Single port
SRAMIE	Single-port robot-assisted minimally invasive esophagectomy
VAE	Video-assisted thoracoscopic esophagectomy

## References

[1] Sung H., Ferlay J., Siegel R.L., Laversanne M., Soerjomataram I., Jemal A., Bray F. (2021). Global Cancer Statistics 2020: GLOBOCAN Estimates of Incidence and Mortality Worldwide for 36 Cancers in 185 Countries. CA A Cancer J. Clin..

[2] Morgan E., Arnold M., Gini A., Lorenzoni V., Cabasag C.J., Laversanne M., Vignat J., Ferlay J., Murphy N., Bray F. (2023). Global burden of colorectal cancer in 2020 and 2040: Incidence and mortality estimates from GLOBOCAN. Gut.

[3] Sheikh M., Roshandel G., McCormack V., Malekzadeh R. (2023). Current Status and Future Prospects for Esophageal Cancer. Cancers.

[4] Obermannova R., Alsina M., Cervantes A., Leong T., Lordick F., Nilsson M., van Grieken N.C.T., Vogel A., Smyth E.C., ESMO Guidelines Committee (2022). Oesophageal cancer: ESMO Clinical Practice Guideline for diagnosis, treatment and follow-up. Ann. Oncol..

[5] Watanabe M., Otake R., Kozuki R., Toihata T., Takahashi K., Okamura A., Imamura Y. (2020). Recent progress in multidisciplinary treatment for patients with esophageal cancer. Surg. Today.

[6] Cuschieri A. (1993). Endoscopic subtotal oesophagectomy for cancer using the right thoracoscopic approach. Surg. Oncol..

[7] Mariette C., Markar S., Dabakuyo-Yonli T.S., Meunier B., Pezet D., Collet D., D’Journo X.B., Brigand C., Perniceni T., Carrere N. (2020). Health-related Quality of Life Following Hybrid Minimally Invasive Versus Open Esophagectomy for Patients With Esophageal Cancer, Analysis of a Multicenter, Open-label, Randomized Phase III Controlled Trial: The MIRO Trial. Ann. Surg..

[8] Straatman J., van der Wielen N., Cuesta M.A., Daams F., Roig Garcia J., Bonavina L., Rosman C., van Berge Henegouwen M.I., Gisbertz S.S., van der Peet D.L. (2017). Minimally Invasive Versus Open Esophageal Resection: Three-year Follow-up of the Previously Reported Randomized Controlled Trial: The TIME Trial. Ann. Surg..

[9] Dantoc M.M., Cox M.R., Eslick G.D. (2012). Does minimally invasive esophagectomy (MIE) provide for comparable oncologic outcomes to open techniques? A systematic review. J. Gastrointest. Surg..

[10] Nuytens F., Dabakuyo-Yonli T.S., Meunier B., Gagniere J., Collet D., D’Journo X.B., Brigand C., Perniceni T., Carrere N., Mabrut J.Y. (2021). Five-Year Survival Outcomes of Hybrid Minimally Invasive Esophagectomy in Esophageal Cancer: Results of the MIRO Randomized Clinical Trial. JAMA Surg..

[11] Kernstine K.H., DeArmond D.T., Karimi M., Van Natta T.L., Campos J.H., Yoder M.R., Everett J.E. (2004). The robotic, 2-stage, 3-field esophagolymphadenectomy. J. Thorac. Cardiovasc. Surg..

[12] Towe C.W., Servais E.L., Brown L.M., Blasberg J.D., Mitchell J.D., Worrell S.G., Seder C.W., David E.A. (2024). The Society of Thoracic Surgeons General Thoracic Surgery Database: 2023 Update on Outcomes and Research. Ann. Thorac. Surg..

[13] Park B.J., Flores R.M., Rusch V.W. (2006). Robotic assistance for video-assisted thoracic surgical lobectomy: Technique and initial results. J. Thorac. Cardiovasc. Surg..

[14] Kang C.H. (2021). Totally Robotic Esophagectomy. J. Chest Surg..

[15] Lee J.H., Park T.H., Kim H.K. (2024). Robotic thoracic surgery using the single-port robotic system: Initial experience with more than 100 cases. J. Thorac. Cardiovasc. Surg..

[16] Lee J.H., Hong J.I., Jin Jang Y., Kim H.K. (2023). Single-port plus one port subcostal robotic-assisted minimally invasive McKewon esophagectomy using the daVinci Single-Port surgical system. Interdiscip. CardioVascular Thorac. Surg..

[17] Mann C., Berlth F., Hadzijusufovic E., Lang H., Grimminger P.P. (2020). Minimally invasive esophagectomy: Clinical evidence and surgical techniques. Langenbeck’s Arch. Surg..

[18] Yun J.K., Lee I.S., Gong C.S., Kim B.S., Kim H.R., Kim D.K., Park S.I., Kim Y.H. (2019). Clinical utility of robot-assisted transthoracic esophagectomy in advanced esophageal cancer after neoadjuvant chemoradiation therapy. J. Thorac. Dis..

[19] Rice T.W., Ishwaran H., Ferguson M.K., Blackstone E.H., Goldstraw P. (2017). Cancer of the Esophagus and Esophagogastric Junction: An Eighth Edition Staging Primer. J. Thorac. Oncol..

[20] Clavien P.A., Barkun J., de Oliveira M.L., Vauthey J.N., Dindo D., Schulick R.D., de Santibanes E., Pekolj J., Slankamenac K., Bassi C. (2009). The Clavien-Dindo classification of surgical complications: Five-year experience. Ann. Surg..

[21] Low D.E., Alderson D., Cecconello I., Chang A.C., Darling G.E., D’Journo X.B., Griffin S.M., Holscher A.H., Hofstetter W.L., Jobe B.A. (2015). International Consensus on Standardization of Data Collection for Complications Associated With Esophagectomy: Esophagectomy Complications Consensus Group (ECCG). Ann. Surg..

[22] National Health Commission of The People’s Republic of China (2022). National guidelines for diagnosis and treatment of esophageal carcinoma 2022 in China (English version). Chin. J. Cancer Res..

[23] Park B.J., Kim D.J. (2021). Robot-Assisted Thoracoscopic Esophagectomy with Total Mediastinal Lymphadenectomy: A Guide to a Systematic Approach Using the Concept of Fascial Plane Dissection. J. Chest Surg..

[24] Harrington C., Molena D. (2021). Minimally invasive Ivor Lewis esophagectomy in 10 steps. JTCVS Tech..

[25] Shimada Y., Okumura T., Nagata T., Sawada S., Matsui K., Hori R., Yoshioka I., Yoshida T., Osada R., Tsukada K. (2011). Usefulness of blood supply visualization by indocyanine green fluorescence for reconstruction during esophagectomy. Esophagus.

[26] You J., Zhang H., Li W., Dai N., Lu B., Ji Z., Zhuang H., Zheng Z. (2022). Intrathoracic versus cervical anastomosis in esophagectomy for esophageal cancer: A meta-analysis of randomized controlled trials. Surgery.

[27] McLarty A.J., Deschamps C., Trastek V.F., Allen M.S., Pairolero P.C., Harmsen W.S. (1997). Esophageal resection for cancer of the esophagus: Long-term function and quality of life. Ann. Thorac. Surg..

[28] Ercan S., Rice T.W., Murthy S.C., Rybicki L.A., Blackstone E.H. (2005). Does esophagogastric anastomotic technique influence the outcome of patients with esophageal cancer?. J. Thorac. Cardiovasc. Surg..

[29] Williams V.A., Watson T.J., Zhovtis S., Gellersen O., Raymond D., Jones C., Peters J.H. (2008). Endoscopic and symptomatic assessment of anastomotic strictures following esophagectomy and cervical esophagogastrostomy. Surg. Endosc..

[30] Aiolfi A., Sozzi A., Bonitta G., Lombardo F., Cavalli M., Cirri S., Campanelli G., Danelli P., Bona D. (2022). Linear- versus circular-stapled esophagogastric anastomosis during esophagectomy: Systematic review and meta-analysis. Langenbecks Arch. Surg..

[31] Kondra J., Ong S.R., Clifton J., Evans K., Finley R.J., Yee J. (2008). A change in clinical practice: A partially stapled cervical esophagogastric anastomosis reduces morbidity and improves functional outcome after esophagectomy for cancer. Dis. Esophagus.

[32] Yang Y., Li B., Yi J., Hua R., Chen H., Tan L., Li H., He Y., Guo X., Sun Y. (2022). Robot-assisted Versus Conventional Minimally Invasive Esophagectomy for Resectable Esophageal Squamous Cell Carcinoma: Early Results of a Multicenter Randomized Controlled Trial: The RAMIE Trial. Ann. Surg..

[33] Chao Y.K., Li Z., Jiang H., Wen Y.W., Chiu C.H., Li B., Shang X., Fang T.J., Yang Y., Yue J. (2024). Multicentre randomized clinical trial on robot-assisted versus video-assisted thoracoscopic oesophagectomy (REVATE trial). Br. J. Surg..

[34] Chen Z., Jiang L., Zheng H., Zhang W., Lv X., Abdellateef A. (2022). Early postoperative pain after subxiphoid uniportal thoracoscopic major lung resection: A prospective, single- blinded, randomized controlled trial. Interdiscip. CardioVascular Thorac. Surg..

[35] Luo Y., He F., Wu Q., Shi H., Chen D., Tie H. (2022). Feasibility of Video-Assisted Thoracoscopic Surgery via Subxiphoid Approach in Anterior Mediastinal Surgery: A Meta-Analysis. Front. Surg..

[36] Bagan P., Das-Neves-Pereira J.C., Abdesselam A.B., Couffinhal J.C. (2010). Intercostal-subcostal combined complete port-accessed video-assisted lobectomy. Interdiscip. CardioVascular Thorac. Surg..

[37] Tagkalos E., Goense L., Hoppe-Lotichius M., Ruurda J.P., Babic B., Hadzijusufovic E., Kneist W., van der Sluis P.C., Lang H., van Hillegersberg R. (2020). Robot-assisted minimally invasive esophagectomy (RAMIE) compared to conventional minimally invasive esophagectomy (MIE) for esophageal cancer: A propensity-matched analysis. Dis. Esophagus.

[38] Zheng C., Li X.K., Zhang C., Zhou H., Ji S.G., Zhong J.H., Xu Y., Cong Z.Z., Wang G.M., Wu W.J. (2021). Comparison of short-term clinical outcomes between robot-assisted minimally invasive esophagectomy and video-assisted minimally invasive esophagectomy: A systematic review and meta-analysis. J. Thorac. Dis..

[39] Suda K., Ishida Y., Kawamura Y., Inaba K., Kanaya S., Teramukai S., Satoh S., Uyama I. (2012). Robot-assisted thoracoscopic lymphadenectomy along the left recurrent laryngeal nerve for esophageal squamous cell carcinoma in the prone position: Technical report and short-term outcomes. World J. Surg..

[40] Oshikiri T., Goto H., Horikawa M., Urakawa N., Hasegawa H., Kanaji S., Yamashita K., Matsuda T., Nakamura T., Kakeji Y. (2021). Incidence of Recurrent Laryngeal Nerve Palsy in Robot-Assisted Versus Conventional Minimally Invasive McKeown Esophagectomy in Prone Position: A Propensity Score-Matched Study. Ann. Surg. Oncol..

[41] Guerra F., Vegni A., Gia E., Amore Bonapasta S., Di Marino M., Annecchiarico M., Coratti A. (2018). Early experience with totally robotic esophagectomy for malignancy. Surgical and oncological outcomes. Int. J. Med. Robot. Comput. Assist. Surg..

[42] Davakis S., Charalabopoulos A., Kyros E., Sakarellos P., Tsourouflis G., Dimitroulis D., Nikiteas N. (2022). Minimally Invasive Transcervical Esophagectomy with Mediastinal Lymphadenectomy for Cancer. A Comparison with Standardized Techniques. Anticancer Res..

[43] Hadzijusufovic E., Lozanovski V.J., Griemert E.V., Bellaio L., Lang H., Grimminger P.P. (2024). Single-Port da Vinci Robot-Assisted Cervical Esophagectomy: How to Do It. J. Thorac. Cardiovasc. Surg..

